# Surgical outcomes of familial exudative vitreoretinopathy-associated retinal detachment: a systematic review and meta-analysis

**DOI:** 10.1186/s40942-026-00850-1

**Published:** 2026-04-11

**Authors:** Hashem Abu Serhan, Ahmad Alazzam, Ahmad Barakat, Anas Alamoudi, Raed Alnutaifi, Nagi Ahmed, Taima’a Al-Awaysheh, Mohd Salim Harun Gigani, Ahmed B. Sallam

**Affiliations:** 1 Department of Ophthalmology, Hamad Medical Corporation, Doha, Qatar; 2 Faculty of Medicine, The Hashemite University, Zarqa, Jordan; 3 Department of Ophthalmology, Jeddah Eye Hospital, Jeddah, Saudi Arabia; 4 College of Medicine, King Saud University, Riyadh, Saudi Arabia; 5 Department of Ophthalmology, Jordanian Royal Medical Services, Amman, Jordan; 6 Department of Ophthalmology, Jones Eye Institute, University of Arkansas for Medical Sciences, 4301 W. Markham Street, Little Rock, AR 72207 USA

**Keywords:** Familial exudative vitreoretinopathies, Rhegmatogenous retinal detachment, Tractional retinal detachment, Pediatric retinal detachment

## Abstract

**Background:**

Familial exudative vitreoretinopathy (FEVR) is a rare hereditary vitreoretinal disorder in children, characterized by abnormal peripheral retinal vascularization leading to retinal detachment (RD) in 21% to 64% of cases. Optimal surgical management remains uncertain owing to disease rarity and heterogeneity of published evidence.

**Purpose:**

To evaluate anatomical and functional outcomes of surgical repair for FEVR-associated RD.

**Methods:**

This PRISMA-adherent systematic review and meta-analysis was prospectively registered in PROSPERO (CRD420251071825). Eligible designs included retrospective and prospective cohort studies and case series reporting surgical outcomes in FEVR-associated RD with more than five patients. Pre- and postoperative best-corrected visual acuity (BCVA; logMAR) was pooled using random-effects models. Retinal reattachment rates were synthesized as proportions. Subgroup analyses were performed by detachment type (rhegmatogenous (RRD) versus tractional (TRD)) and surgical approach (pars plana vitrectomy (PPV), scleral buckling (SB), combined PPV + SB). Heterogeneity was quantified using I² and 95% prediction intervals. Formal meta-regression was not performed, given insufficient study counts per subgroup.

**Results:**

Twenty studies comprising 586 eyes were analyzed. Overall surgery yielded a significant BCVA improvement (MD -0.30 logMAR; 95% CI -0.50 to -0.08; *p* = .009) with substantial heterogeneity (I² = 86%; prediction interval − 1.03 to 0.45). RRD demonstrated greater functional benefit (MD -0.45 logMAR; 95% CI -0.72 to -0.16) and high anatomical success (88.8%; I² = 0%). TRD showed no significant visual improvement (MD -0.15; *p* = .38) and lower, more variable reattachment (72.8%; I² = 63.3%). SB achieved the highest reattachment rate (93.2%), combined PPV + SB the most consistent gains (84.0%; I² = 0%), and PPV alone the lowest reattachment (63.0%), reflecting its preferential use in complex TRD. FEVR staging was inconsistently reported, precluding stage-stratified analysis.

**Conclusion:**

Surgical repair of FEVR-associated RD yields meaningful outcomes, particularly in RRD, while TRD goals are centered on vision preservation. Findings are limited by retrospective designs, substantial heterogeneity, and inconsistent staging data. Prospective multicenter registries with standardized reporting are urgently needed.

**Supplementary Information:**

The online version contains supplementary material available at 10.1186/s40942-026-00850-1.

## Introduction

Familial Exudative Vitreoretinopathy (FEVR) is a hereditary retinal disease that mainly affects children and is characterized by differentperipheral retinal vascular anomalies, such as nonperfusion, telangiectasias, aneurysms, and dragging or straightening of the vasculature. Retinal detachment, vitreoretinal traction, vitreous hemorrhage, and neovascularization are all consequences of nonperfusion, which result in major vision loss or blindness [1].

FEVR follows an autosomal dominant, autosomal recessive, or X-linked inheritance pattern, although sporadic cases with no identifiable family history have been reported [2]. Causative mutations have been identified in several genes regulating the Norrin-Frizzled4 signaling pathway, including FZD4, LRP5, TSPAN12, NDP, and CTNNB1, with NDP and FZD4 mutations most strongly associated with severe phenotypes and retinal detachment risk [3]. In 1998, Pendergast and Trese^3^ introduced a five-stage grading system that remains the most widely used classification in clinical practice. Stages 1 and 2 denote peripheral avascularity and extraretinal neovascularization without detachment; stage 3 describes subtotal retinal detachment not involving the macula; and stages 4 and 5 represent subtotal and total retinal detachment with macular involvement, respectively, each further subdivided by the presence of exudation. Surgical prognosis deteriorates markedly with advancing stage, with reported anatomical success rates of approximately 70% at stage 4 and 45% at stage 5 [4]. 

Patients are often asymptomatic in the early stages, but may show evidence of peripheral retinal avascularity in more severe stages. Patients may experience decreased visual acuity due to neovascularization, subretinal exudation, or retinal detachment. Moreover, Leukocoria might be the first presentation for a child in cases of total retinal detachment or extensive exudate [5]. 

FEVR can lead to tractional, exudative, or rhegmatogenous retinal detachment (RRD), which occurs in 21% to 64% of FEVR individuals [6]. Among these, Tractional retinal detachment (TRD) is the predominant phenotype, while exudative RD appears less common and often arises earlier in life. In contrast, little is currently known about FEVR-associated RRD [7]. 

The management of RD associated with FEVR is complex and varies significantly based on several factors such as the type of RD, stage of the disease, age of the patient, and the presence of additional ocular abnormalities. The treatment involves different surgical methods, which include vitreoretinal surgery techniques like pars plana vitrectomy (PPV), scleral buckling (SB), or combined procedures and laser photocoagulation. The success of these interventions depends on the correct timing because it determines both anatomical and functional results [8] with poor prognosis in advanced stages, and success rates of 70% in stage 4 and 45% in stage 5 [9].

The literature about surgical management of FEVR and RD has expanded, yet researchers have not developed a systematic method to assess the effectiveness of these surgical interventions. The results of these treatments show variability between studies because of different ways of classifying RD and different surgical methods, and the subjective nature of reporting outcomes [10, 11].

This systematic review and meta-analysis will combine existing studies about the surgical treatment of FEVR-associated RD. The primary objective of this systematic review and meta-analysis was to evaluate the change in best-corrected visual acuity (BCVA) following surgical repair of FEVR-associated RD. The secondary objective was to determine the pooled rate of anatomical reattachment, with subgroup analyses by detachment type (RRD vs. TRD) and surgical approach (PPV, SB, combined PPV + SB) to provide valuable insights that can guide clinical practice, improve patient outcomes, and identify gaps in current research.

## Materials and methods

We conducted this review in line with the tenets of the Declaration of Helsinki (version 2013) [12]. Our study adhered to the guidelines outlined in the Cochrane Handbook for Systematic Reviews of Interventions and PRISMA guidelines [13]. We followed the Ethical integrity of ophthalmic evidence synthesis [14]. The necessity for institutional review board (IRB) approval was not required since it did not involve human subjects. The study protocol was registered prospectively in PROSPERO under (CRD420251071825).

### Literature search strategy

In June 2025, we conducted a comprehensive search on PubMed, Scopus, Web of Science (WOS), Embase, Google Scholar, Cochrane Reviews, Clinical Trial.gov, and ProQuest Dissertations & Theses covering articles published until June 2025, with no restrictions on publication date applied, as older case series represent important and irreplaceable evidence in this rare condition. We employed the Medical Subject Headings (MeSH) and free-text search terms to identify relevant studies: “Familial Exudative Vitreoretinopathy” [MeSH], “Retinal Detachment” [MeSH], “Rhegmatogenous Retinal Detachment” [MeSH], “Vitrectomy” [MeSH], “Scleral Buckling” [MeSH], and “Retinal Reattachment” [MeSH]. The detailed search strategy for each database is provided in Supplementary Table 11. Notably, we performed a final database search immediately before data analysis to capture any recently published studies, yielding no additional results. Following screening, we conducted a supplementary manual by (1) exploring PubMed’s “similar articles” for each included study, (2) reviewing reference lists of included articles, and (3) performing a Google search using the original keywords.

## Inclusion and exclusion criteria

We followed the population, intervention, comparator, outcomes, and study design (PICOS) to define study eligibility [15]. We included studies that met the following criteria: participants were patients of any age or sex diagnosed with FEVR–associated RD, confirmed by clinical examination and/or genetic testing; interventions involved any surgical approach, including PPV, SB, combined PPV/SB, and the use of adjunctive tamponades such as gas or silicone oil; comparator groups were not required; outcomes included change in best-corrected visual acuity (BCVA), anatomical reattachment rate at the last follow-up, and complication rates; and eligible study designs included retrospective or prospective cohort studies, interventional studies, and case series with more than five patients that reported surgical outcomes in FEVR-associated RD. No minimum follow-up duration was specified as an eligibility criterion; outcomes were extracted at the last postoperative assessment reported in each study.

We excluded studies that did not meet these predefined criteria, including case reports with fewer than five patients, non-English articles, reviews, commentaries, editorials, and studies involving mixed etiologies in which FEVR-associated RD outcomes could not be extracted separately.

## Screening and study selection

We uploaded articles retrieved through the systematic search to the Rayyan website (https://www.rayyan.ai/), where duplicates were determined and removed. Two independent authors [NA and RA] screened the titles and abstracts of the search results for relevance, followed by full-text screening, and all authors agreed on the final list of included studies. We solved the disagreement among reviewers through consensus.

## Data extraction

We used a standardized extraction Excel (Microsoft, USA) sheet to collect relevant data from each included study. For study-level characteristics, we recorded the study ID (first author and year), publication year, country, study design (prospective or retrospective), sample size in eyes and patients, inclusion and exclusion criteria, and the duration of follow-up. For patient-level characteristics, we included the number and proportion of eyes with RRD and TRD, lens status, surgical techniques performed PPV, SB, combined PPV and SB, final anatomical reattachment rate, and BCVA expressed in LogMAR units (preoperative and postoperative means with standard deviations). In studies reporting visual acuity using semi-quantitative categories such as counting fingers (CF) and hand motion (HM), values were converted to LogMAR equivalents based on previously validated estimates derived from the Freiburg Visual Acuity Test (CF ≈ 1.98 logMAR; HM ≈ 2.28 logMAR). In studies reporting individual participant data (IPD), we extracted de-identified, eye-level data including study ID, preoperative and postoperative BCVA (LogMAR), and binary retinal reattachment outcome. We used these IPD records for additional pooled analyses and allowed estimation of continuous outcomes such as visual improvement and individual-level reattachment probabilities. We did not analyze subgroups with fewer than to ensure stability of effect estimates. Two independent reviewers [AA and AA] performed data extraction. We solved disagreements among reviewers through consensus consultation with the senior author [HAS]. Individual patient data (IPD) were extracted from published eye-level data tables where available; no requests were made to authors for unpublished datasets.

### Study outcomes

The primary outcome was the change in BCVA, expressed in logMAR units, from baseline to final postoperative assessment. BCVA was treated as a continuous outcome. For each study, the effect size was defined as the mean difference in BCVA (postoperative logMAR minus preoperative logMAR), with negative values indicating improvement [16]. The secondary outcome was the rate of retinal reattachment at final follow-up. Retinal reattachment was defined as complete anatomical reattachment of the retina at the last postoperative visit, as reported by the study authors. Partial reattachment or cases requiring additional surgery were not counted as successfully reattached. Retinal reattachment was treated as a dichotomous outcome (yes/no) in the analysis, extracted as the proportion of eyes achieving complete reattachment.

### Statistical analysis

We conducted a meta-analysis to evaluate the change in BCVA. The effect size was defined as the mean difference (MD) between postoperative and preoperative logMAR values, with negative values indicating improvement. and to calculate the retinal reattachment rate as a proportion. To estimate the standard error (SE) of the paired difference, we assumed a correlation coefficient (r) of 0.5 between pre- and postoperative measurements. We calculated the standard deviation of the difference (SD_diff) using the following formula:

$$S{D_{diff}} = \sqrt {SD_{pre}^2 + SD_{post}^2 - 2r \cdot S{D_{Pre}} \cdot S{D_{post}}}$$

We then calculated the standard error as:$\:SE=\frac{{SD}_{diff}}{\sqrt{n}}$

Where n represents the number of eyes analyzed in each study.

We performed a random-effects meta-analysis using the restricted maximum likelihood (REML) estimator and applied Knapp–Hartung adjustments to compute confidence intervals. We quantified between-study heterogeneity using the I² statistic and τ² and reported the 95% prediction intervals (PIs). Because I² can be unreliable in small meta-analyses and does not capture the clinical dispersion of effects, we interpreted it with caution. Similarly, Cochran’s Q has limited power to detect true between-study variability.

Sensitivities were conducted for both primary and secondary outcomes, including:

(1) variation of the assumed pre–post correlation (*r* = .3, 0.5, 0.7) for BCVA outcomes;

(2) leave-one-out (LOO) analyses applied to all pooled analyses (overall and subgroup analyses for BCVA and reattachment outcomes) to assess the influence of individual studies; and.

(3) outlier analyses, in which studies were excluded if they demonstrated extreme effect sizes and/or were identified as major contributors to heterogeneity (based on influence diagnostics and inspection of forest plots). Publication bias was assessed using Egger’s regression test for outcomes with ≥ 10 studies [17]. 

All statistical analyses were performed in R (version 4.3.2) using the meta and metafor packages. Forest plots were generated using the forestplot package.

### Quality assessment

We used the Newcastle-Ottawa scale (NOS) for quality assessment of non-randomized studies for the quality assessment of the included studies [18]. The NOS assigns a maximum of nine points for the three domains: (1) Selection of study groups (four points); (2) Comparability of groups (two points); and 3) Ascertainment of exposure and outcomes (three points). NOS’s total score of 0 to 3 indicates a high risk of bias, 4 to 6 indicates a moderate risk, and ≥ 7 indicates a low risk of bias.

## Results

### Search selection

**Fig. 1:**
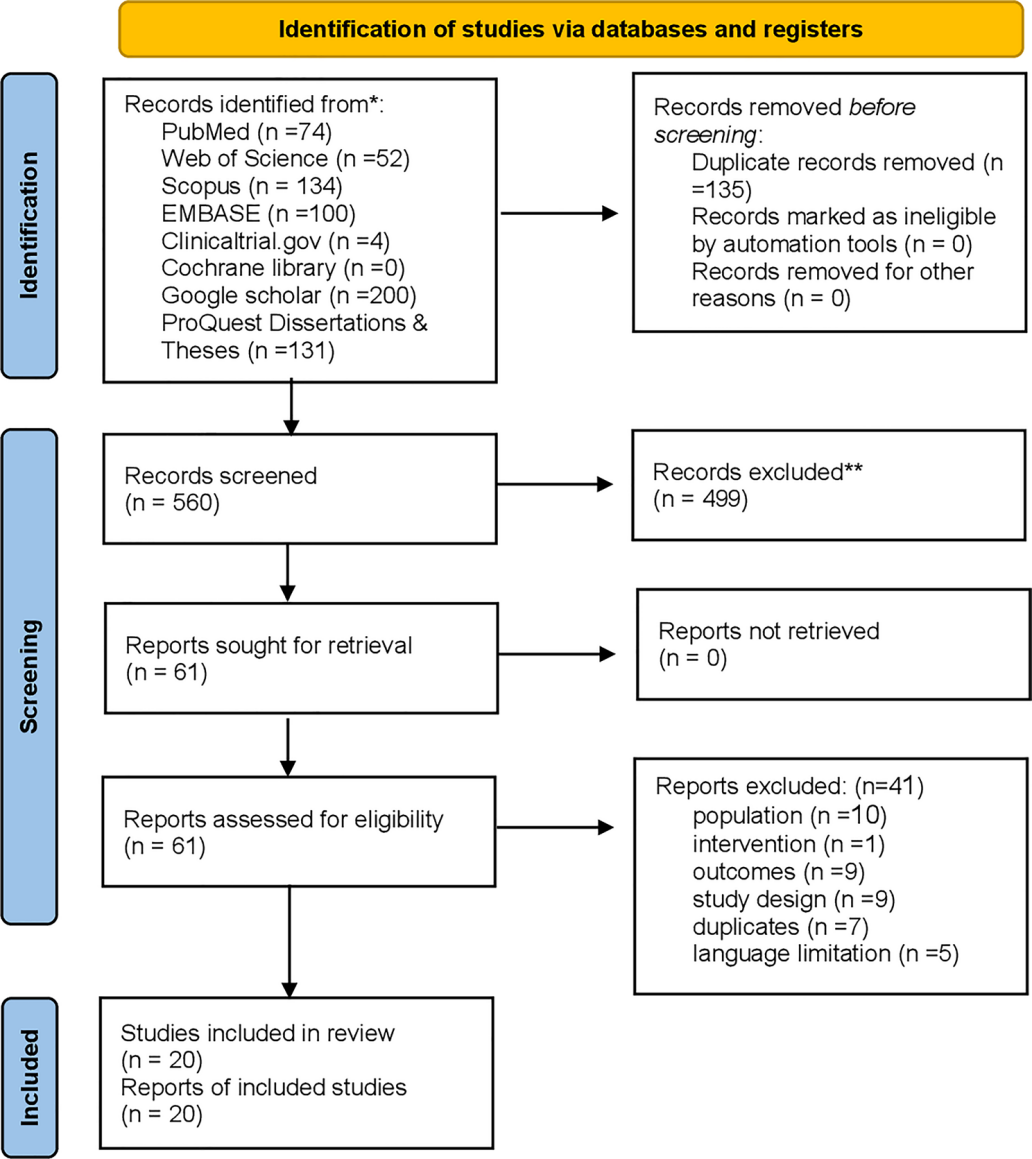
PRISMA flow diagram of study selection

A comprehensive search across eight databases yielded 695 records: PubMed (*n* = 74), Web of Science (*n* = 52), Scopus (*n* = 134), EMBASE (*n* = 100), ClinicalTrials.gov (*n* = 4), Cochrane Library (*n* = 0), Google Scholar (*n* = 200), and ProQuest Dissertations & Theses (*n* = 131). After removing 135 duplicates, we screened 560 unique records by title and abstract. We excluded 499 records and assessed 61 full-text articles for eligibility. We further excluded 40 studies after full-text review for the following reasons: inappropriate population (*n* = 10), irrelevant outcomes (*n* = 9), unsuitable study design (*n* = 9), previously unidentified duplicates (*n* = 7), non-English language (*n* = 5), and intervention mismatch (*n* = 1). Eligible for final analysis were 20 studies in the final qualitative and quantitative synthesis, as illustrated in the PRISMA flowchart. (Fig. 1)

### Baseline studies’ characteristics

A total of 20 [4, 9, 11, 19–34] Studies were included, encompassing 586 eyes diagnosed with FEVR. The studies, published between 1995 and 2025, were conducted across diverse geographic regions, including Asia (India, Japan, China, Taiwan, and Turkey) [11, 19–22, 24, 25, 27, 28, 30, 33, 35], Europe (France) [9], and North America (United States) [5, 23, 26, 34]. Most were retrospective case series (*n* = 15), with the remainder comprising prospective observational (*n* = 2), prospective interventional (*n* = 1), and retrospective cohort (*n* = 2) designs. Mean Follow-up durations ranged widely, from 7 months [27]. to 216 months [29] as detailed in Table 1.

**Table 1:** Characteristics of the included studies

Study ID	Year	Country	Study design	Inclusion criteria	Exclusion criteria	Follow-up period
Agrawal 2022	2022	India	Prospective interventional study	(1) Diagnosis of FEVR (as per specific angiographic and clinical findings) (2) Macula-off RRD (3) Age ≤ 18 years	(1) Prior history of intraocular surgery (2) Uncertain diagnosis of FEVR	Mean 3.32 ± 1.34 years (range: 1.5–5 years)
Katagiri 2017	2017	Japan	Retrospective, noncomparative case series	Patients with clinically diagnosed FEVR and suspected Rhegmatogenous retinal detachment (RRD)	Missing data- RRD with radial retinal folds due to complexity in classification	5.8 ± 4.8 years (Range: 0.3 to 21.3 years)
Chen et al., 2012	2012	Taiwan	Retrospective interventional case series	Diagnosis of FEVR confirmed by fluorescein angiography showing peripheral avascularity; presence of RRD with identifiable retinal breaks	Premature birth, incubator history, systemic diseases (e.g., uveitis, collagen vascular disease)	January 2002 to March 2009 (mean follow-up period not explicitly stated)
Huang et al., 2022	2022	China	Retrospective interventional case series	Patients with FEVR-associated Rhegmatogenous retinal detachment (FEVR-RRD) who underwent encircling scleral buckling (ESB) surgery with cryotherapy	Not explicitly mentioned	Mean 34.5 ± 27.7 months (range 7–104 months)
Oga 2025	2025	Japan	Retrospective chart review	TRD associated with FEVR; surgery between 2011–2022; follow-up ≥ 18 months	Rhegmatogenous RD, ROP, PFV, Coats’ disease, ocular toxocariasis	Median 55.1 months (range 18–144)
Fei 2016	2016	China	Retrospective case series	Advanced FEVR (Stage 3+) with complications (retinal detachment, corneal opacity, shallow/flat anterior chamber, cataract, glaucoma, etc.)	Norrie disease, PHPV, retinopathy of prematurity, or other ocular diseases	12–46 months (mean not specified)
Peng 2022	2022	China	Retrospective case series	Stage 5 FEVR with anterior segment anomalies (e.g., shallow AC, capsule-endothelial adhesion, secondary glaucoma)	Phthisis bulbi, keratoleukoma > 2/3 corneal area	Mean 13.9 ± 5.2 months
Zou 2024	2024	China	Retrospective case series	Stage 5 FEVR with funneled RD; IRBs during vitrectomy; cyanoacrylate glue application	Obvious neovascularization, vitreous hemorrhage, Norrie’s disease, or unclear diagnosis	Mean 12.5 months (range 9.8–18.8
Glazer 1995	1995	USA	Retrospective case series	Children (18 months–9 years) with FEVR and traction retinal detachment requiring vitrectomy.	Not explicitly stated; presumed exclusion of non-FEVR retinal disorders (e.g., ROP, Coats’ disease).	Mean 21 months (range: 12–36 mo)
Liu 2024	2024	China	Retrospective case series	Stage 3 or 4 FEVR with tractional RD, treated with lens-sparing vitrectomy (LSV). Full-term birth, no prematurity.	Prematurity, birth weight < 1,250 g, skin lesions, or telangiectasias.	Median 21 months (range: 3–105 mo)
Yamane 2014	2014	Japan	Retrospective case series	FEVR with progressive TRD; active fibrovascular proliferation	Cicatricial total RD, Rhegmatogenous RD	Mean 52 months (range 17–112)
Ma 2025	2025	China	Retrospective case series	Children < 14 years with FEVR and tractional maculopathy requiring vitrectomy with preretinal membrane peeling.	(1) Follow-up < 3 months; (2) Other ocular diseases/trauma; (3) Inadequate imaging data.	Mean 14.1 months (range: 3–40 mo)
Ma 2018	2018	China	Prospective observational	Infants with FEVR, retrolental adhesion, macular detachment within retinal fold, partial lens opacity (≤ 2/3 of pupil)	Questionable FEVR diagnosis; prior vitrectomy/scleral buckle; Norrie’s disease/ROP; dense cataracts	7 months
el-Khoury 2020	2020	France	retrospective	Age less than 18 years underwent PPV for FEVR, minimum follow up of 6 months	Other diseases with similar clinical features to FEVR.	Mean 3.3Y ± 3.4 Range 0.5–15.7 Y
Hocaoglu 2016	2016	turkey	retrospective	advanced FEVR requiring PPV with a Minimum follow up of 12 M	Other diseases with similar clinical features to FEVR.	Mean 58.4 ± 75.1 M Range 12–216 M
Sen 2020	2020	India	Retrospective	Final diagnosis of FEVR (full term, lack of peripheral retinal vascular development in at least one eye, variable degrees of vitreoretinal traction or subretinal exudation or retinal neovascularization)	Not explicitly mentioned	Mean 22.7 ± 19.4 M Range 1.8 M – 7.2Y
Ikeda 1999	1999	japan	retrospective	Clinically diagnosed FEVR	Not explicitly mentioned	Mean 25.8 ± 11.8 M Range 12–49 M
Shubert 1997	1997	USA	retrospective	FEVR surgery due to vitreous hemorrhage and/or TRD	Not explicitly mentioned	Mean 6.4 Y Range 1-26.25 Y
Hubbard 2021	2021	United States	Retrospective cohort	Patients ≤ 21 years of age with a diagnosis of FEVR who underwent laser and/or anti-VEGF treatment at the study centers between 2001–2018	Follow-up < 3 months - Age > 21 years at presentation or treatment - Inaccessible records - Treated only at outside facilities	Mean 57.8 months (Range: 6.6–134 months)
Pendergast 1998	1998	USA	retrospective	A diagnosis of FEVR based on characteristic fundus findings and birth history	Not explicitly mentioned	Mean 35.2 ± 24.0 moths

### Baseline patients’ characteristics

Across 20 studies, 16 studies reported individual-patient data (IPD) [9, 11, 19, 20, 22, 26, 28–30, 34] and 4 aggregate datasets [21, 27, 31, 33] Comprising 586 eyes, the median study mean age was 3.7 years study means ranged from 0.7 [27] to 18.8 years [19] and the average proportion of male participants across studies was 61.1%. Detachment type was reported for 428 eyes (170 rhegmatogenous, 258 tractional); the remaining 158 eyes were not specified by detachment type in four studies [4, 9, 27, 32]. Surgical management included PPV in 377 eyes, SB in 113 eyes, and combined PPV + SB in 96 eyes. Sixteen of 20 studies reported postoperative BCVA, and 19 of 20 reported anatomical reattachment outcomes. Most studies used IPD, and several contributed multiple detachment and surgery types; when the type was not reported at the eye level, those eyes were excluded from detachment-type subgroup counts as detailed in Table 2.

**Table 2:** Baseline characteristics of the included eyes

StudyID	Data Type (AD/IPD)	Male gender (*n*, %)	Mean Age in years (± SD)	Total Eyes	# RRD Eyes	# TRD Eyes	# PPV	# SB	# PPV + SB	BCVA Reported	RRR Reported
Agrawal 2022	IPD	11 (78.6%)	12.1 ± 3.2	14	14	0	10	4	0	Yes	Yes
Chen 2012	IPD	20 (83.3%)	16.4 ± 5.5	24	24	0	0	18	6	Yes	Yes
Fei 2016	IPD	5 (41.7%)	5.8 ± 8.1	12	0	0	12	0	0	Yes	Yes
Glazer 1995	IPD	NR	3.4 ± 1.9	6	0	6	5	0	1	Yes	Yes
Hocaoglu 2016	IPD	4 (66.7%)	10.5 ± 7.5	6	0	0	6	0	0	Yes	Yes
Huang 2022	IPD	16 (66.7%)	14.1 ± 8	24	24	0	0	21	3	Yes	Yes
Hubbard 2021	IPD	4 (50%)	9.7 ± 4.4	8	0	8	5	1	2	Yes	No
Ikeda 1999	IPD	17 (60.7%)	18.8 ± 9.3	28	25	0	0	0	28	Yes	Yes
Ma 2025	IPD	7 (46.7%)	7.2 ± 3.5	15	0	15	14	0	1	Yes	Yes
Pendergast 1998	IPD	15 (57.7%)	3.5 ± 4.7	32	Not Specified		26	4	2	Yes	Yes
Peng 2022	IPD	4 (66.7%)	2.8 ± 1	6	Not Specified		6	0	0	No	Yes
Sen 2020	IPD	25 (57.8%)	14.6 ± 10.9	44	35	9	5	13	26	Yes	Yes
Shubert 1997	IPD	3 (42.9%)	16.5 ± 10.4	7	2	0	3	3	1	Yes	Yes
Yamane 2014	IPD	17 (54.8%)	3.1 ± 4.3	31	0	31	16	12	3	Yes	Yes
el-Khoury 2020	IPD	23 (68%)	5.3 ± 4.8	43	Not Specified		30	0	13	No	Yes
zou2014	IPD	8 (88.9%)	1.6 ± 1	9	0	9	9	0	0	Yes	Yes
Oga2025	AD	32 (68.1%)	4.07 ± 3.35	47	0	47	43	0	4	Yes	Yes
Ma 2018	AD	21 (41%)	0.7 ± 0.15	51	Not Specified		51	0	0	No	Yes
Liu 2024	AD	73 (61.3%)	3.74 ± 2.44	133	0	133	133	0	0	No	Yes
Katagiri 2017	AD	22 (59.5%)	7.1 ± 6.7	46	46	0	3	37	7	Yes	Yes

### Visual acuity

#### Overall visual acuity change

**Fig. 2:**
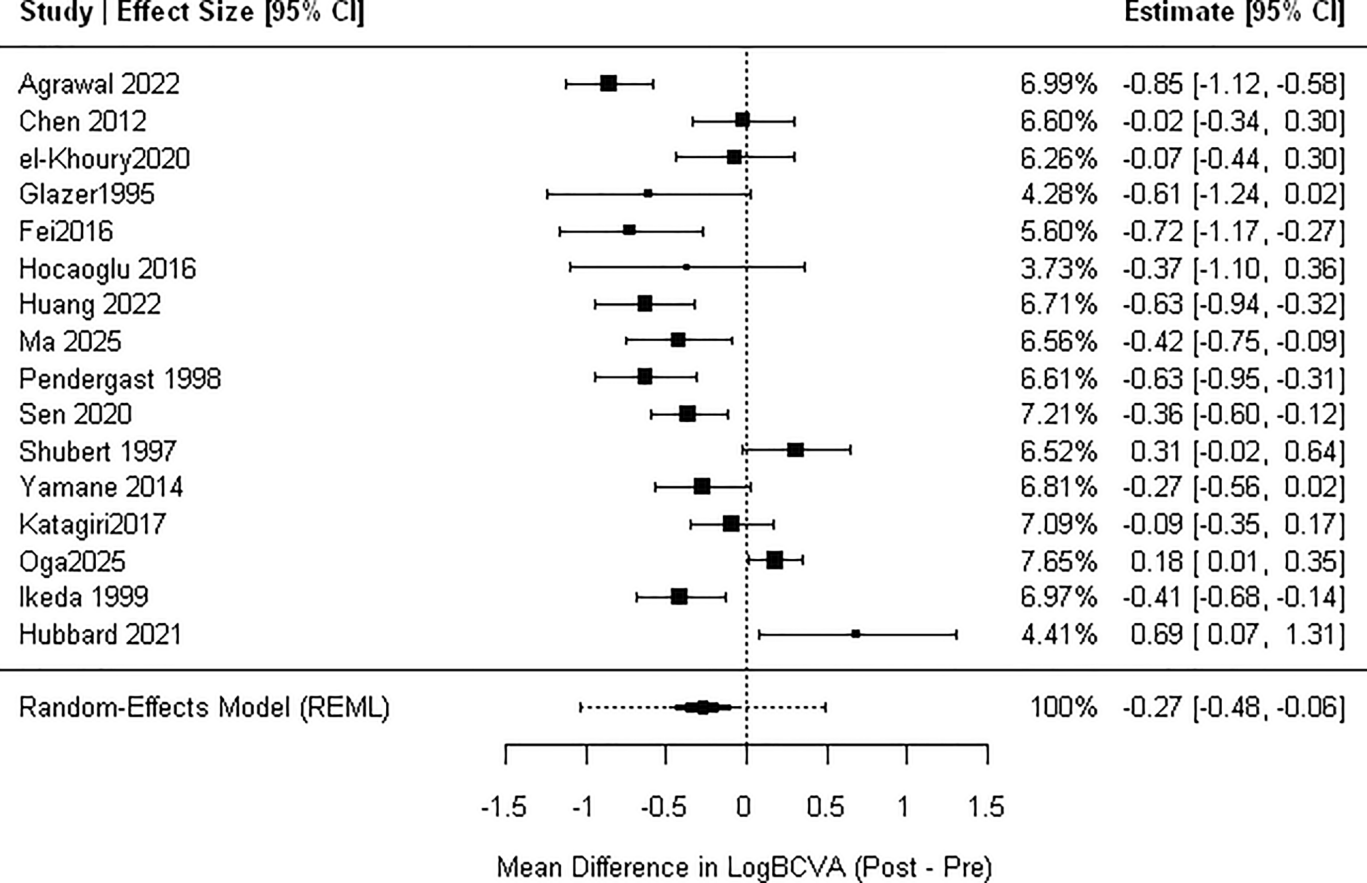
Forest plot of overall BCVA change

Across 16 studies, retinal surgery was associated with a significant improvement in BCVA (MD, − 0.30; 95% CI, − 0.50 to − 0.08; *p* = .009). This corresponds roughly to an improvement of 3 lines on a Snellen chart, although heterogeneity was substantial (I² = 86%; τ² = 0.332). The 95% prediction interval (–1.03 to 0.446). Sensitivity analyses using alternative pre–post correlations produced similar estimates (MD ≈ − 0.27), and LOO analyses confirmed that no single study materially altered the pooled effect as shown in Fig. 2.

Outcomes stratified by detachment type (RRD vs. TRD) demonstrated divergent patterns in both visual and anatomical success, as detailed below.

### Rhegmatogenous retinal detachment (RRD)

Across 6 studies, surgery yielded a pooled mean BCVA improvement of − 0.45 logMAR (95% CI, − 0.72 to − 0.16), with substantial heterogeneity (I² = 72.5%; τ² = 0.05; Q-test *p* = .0002). The 95% prediction interval was wide (–1.08 to 0.19). Sensitivity and LOO analyses confirmed the robustness of these results as shown in Figure S1.

### Tractional retinal detachment (TRD)

Across 7 studies, surgery for TRD was not associated with a significant improvement in BCVA (MD = − 0.15 logMAR; 95% CI, − 0.53 to 0.23; *p* = .38, ~ 1–2 Snellen lines), with substantial heterogeneity (I² = 75%; τ² = 0.103; Q-test *p* = .0004). The 95% prediction interval ranged from 1.02 to 0.73. Sensitivity analyses using different pre–post correlations yielded nearly identical results, and LOO analyses did not materially alter the pooled estimate, as shown in Figure S2.

Stratification by surgical technique further revealed differences in anatomical success rates, most notably lower reattachment rates after PPV compared with SB and combined approaches, likely reflecting case selection for more complex detachments.

### Pars plana vitrectomy (PPV)

Across 9 studies, PPV improved BCVA (MD = − 0.41 logMAR; 95% CI, − 0.69 to − 0.12; *p* = .011; ~4 Snellen lines). with moderate heterogeneity (I² = 68%; τ² = 0.089; Q-test *p* = .0002. Prediction interval: -1.15 to 0.34, as seen in Figure S3. Sensitivity analyses led to the exclusion of the study by Sen 2020)(31), which increased the pooled effect (MD = − 0.51; 95% CI, − 0.78 to − 0.23; ~5 Snellen lines) and reduced heterogeneity (I² = 45%; τ² = 0.044; Q *p* = .079) as illustrated in Figure S4.

Across 5 studies, pooled analysis of LogMAR BCVA after PPV for TRD showed no significant improvement, with a mean difference of − 0.21 (95% CI, − 0.61 to 0.19). Heterogeneity was moderate (I² = 46.8%; τ² = 0.049; Q *p* = .099; ~2 Snellen lines), and the 95% prediction interval ranged from − 0.95 to 0.52, as illustrated in Figure S5.

### Scleral buckling (SB)

Across three studies reporting postoperative BCVA after SB, the pooled mean difference showed a significant improvement of − 0.41 logMAR (95% CI, − 0.74 to − 0.08; *p* = .033; ~4 Snellen lines) and 95% PI ranged from − 0.86 to 0.042, without heterogeneity (I² = 21%; τ² = 0.005; Q *p* = .287) as shown in Figure S6.

### Combined pars plana vitrectomy (PPV) + scleral buckling (SB)

In four studies, combined PPV and SB produced a significant BCVA improvement (MD = − 0.39; 95% CI, − 0.67 to − 0.103; ~4 Snellen lines), with negligible heterogeneity (I² = 0%; τ² ≈ 0; Q-test *p* = .220). LOO confirmed robustness. The 95% prediction interval (–0.672 to − 0.104) is shown in Figure S7. Across 3 studies, the pooled mean difference in LogMAR BCVA after combined PPV and SB for RRD was − 0.41 (95% CI, − 0.878 to 0.04; ~4 Snellen lines), with negligible heterogeneity (I² = 0%; τ² = 0; Q *p* = .338) and a 95% prediction interval of − 0.879 to 0.04 as seen in Figure S8.

### Retinal reattachment rate

#### Overall retinal reattachment rate

**Fig. 3:**
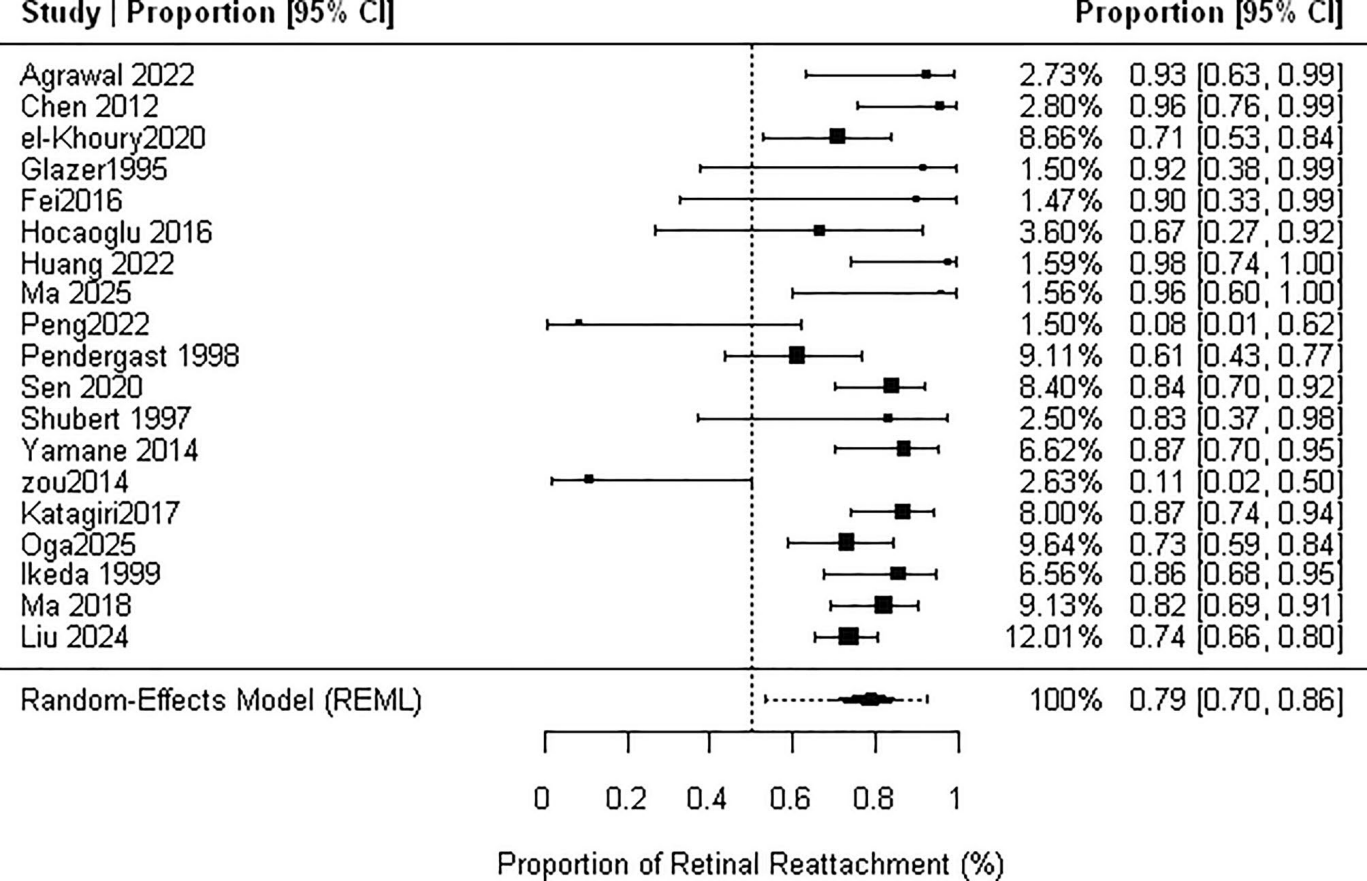
Forest plot of overall retinal reattachment rate (RRR)

Across 19 studies, the pooled retinal reattachment rate was 79.1% (95% CI, 69.6–86.2%) with substantial heterogeneity (I² = 50.4%, τ² = 0.27, *p* for heterogeneity = 0.0015). The 95% prediction interval ranged from 53.4% to 92.6% as shown in Fig. 3. After exclusion of the outlier study(23)The pooled reattachment rate increased to 80.1% (95% CI, 71.4–86.7%) with lower heterogeneity (I² = 34.6%, τ² = 0.15, *p* = .004) and a narrower prediction interval (61.2–91.2%) as shown in Figure S9.

#### Rhegmatogenous retinal detachment (RRD)

Across six studies, anatomical success after RRD surgery was 88.8% (95% CI, 81.7%–93.3%) with negligible heterogeneity (I² = 0%; τ² = 0; Q *p* = .61). Sensitivity analyses confirmed robustness as shown in Figure S10.

#### Tractional retinal detachment (TRD)

Across 10 studies, pooled reattachment after TRD surgery was 72.8% (95% CI, 52.3–86.7%), with substantial heterogeneity (I² = 63.3%, τ² = 0.44, *p* for heterogeneity = 0.0089). The 95% prediction interval was wide (31.8–93.9%) as shown in FigureS11. After exclusion of *Zou 2014* (26)(iatrogenic TRDs) in Figure S12, the pooled reattachment rate increased to 75.8% (95% CI, 67.5–82.6%) with no evidence of heterogeneity (I² = 0%, τ² = 0, *p* = .11), and a narrowed 95% prediction interval (67.5–82.6%),

#### Pars plana vitrectomy (PPV)

Across 10 studies, the pooled proportion of retinal reattachment after PPV was 63.0% (95% CI, 38.9%–82.0%), with moderate heterogeneity (I² = 61.3%; τ² = 0.591; Q *p* = .0102) and a wide 95% prediction interval (18.7%–92.6%) as in Figure S13. Excluding (Zou 2014)(26) reduced heterogeneity (I² = 20.9%; τ² = 0.092) and increased the pooled estimate to 68.5% (95% CI, 53.1%–80.7%), with a narrower prediction interval (45.5%–85.0%) as seen in Figure S14.

In 7 studies with TRD undergoing PPV, the pooled reattachment rate was 57.3% (95% CI, 19.0–88.5; I² = 74.1%) across seven studies, as in Figure S15. Excluding one study with iatrogenic cases (Zou 2014) improved consistency, yielding a reattachment rate of 67.0% (95% CI, 29.5–90.8; I² = 49.1%) as in Figure S16.

#### Scleral buckling (SB)

Across four studies, reattachment after scleral buckle, in which almost all cases were RRD, was 93.2% (95% CI, 72.3%–98.6%) with low heterogeneity (I² = 12.6%; τ² = 0.22; Q *p* = .47). The 95% prediction interval ranged from 59.8% to 99.2% as in Figure S17.

#### Combined pars plana vitrectomy (PPV) + scleral buckling (SB)

In four studies, combined PPV and SB achieved a pooled reattachment rate of 84.0% (95% CI, 62.0%–94.4%) with low heterogeneity (I² = 9.97%; τ² = 0.0587; Q *p* = .364). The 95% prediction interval (56.5%–95.5%) is seen in Figure S18.

Across 3 studies, the pooled retinal reattachment rate after combined PPV and SB for RRD was 86.4% (95% CI, 71.7%–94.1%), with no evidence of heterogeneity (I² = 0%; τ² = 0; Q *p* = .762). The 95% prediction interval ranged from 71.7% to 94.1% as in Figure S19.

### Quality assessment

All included cohort studies were assessed using the NOS, with 11 studies showing a low risk of bias [4, 20–22, 27, 31–34]. However, the rest of the studies were rated as having a moderate risk, as detailed in Supplementary Table 2.

### Publication bias

For the outcomes that contained more than 10 studies, Funnel plot inspection revealed symmetrical distribution of effect sizes. Egger’s test indicated no significant small-study effects as shown in Figures S20 and S21.

## Discussion

This systematic review and meta-analysis of 20 studies (586 eyes) demonstrates that surgical repair of FEVR-associated RD yields meaningful but variable improvements in visual acuity and high rates of anatomical success (~ 80%). These findings are based on predominantly retrospective, observational data, reflecting low-to-very-low certainty of evidence by GRADE criteria, and should therefore be interpreted alongside individual patient and disease characteristics rather than applied prescriptively.

RRD consistently outperformed TRD across both functional and anatomical outcomes. RRD achieved a pooled BCVA improvement of − 0.45 logMAR and 88.8% reattachment with negligible heterogeneity (I² = 0%), results comparable to those reported by Read et al. [36] (84% reattachment, 0.42 logMAR gain) in the broader pediatric RD cohort. TRD, by contrast, showed no significant visual improvement (MD = − 0.15; *p* = .38) and lower, more variable reattachment (72.8%; I² = 63.3%). This VA-anatomy dissociation in TRD is explained by irreversible photoreceptor damage from chronic traction, persistent macular dragging and ectopia despite reattachment, and a high amblyopia burden in this pediatric population [13, 28]. Surgical reattachment in TRD should therefore be framed as vision-preserving rather than vision-restoring, and early intervention — before macular involvement — is likely the single most important prognostic determinant [37]. 

PPV broadly improved BCVA (− 0.41 logMAR) but showed the lowest reattachment rate (63.0%; I² = 61.3%). SB achieved the highest reattachment rate (93.2%) in predominantly RRD cases. Combined PPV + SB offered the most consistent results overall (reattachment 84.0%; BCVA MD − 0.39; I² 0%). The lower PPV reattachment rate reflects case-selection bias rather than technique inferiority: PPV was preferentially applied to complex TRD with fibrovascular proliferation and advanced staging, mirroring patterns in the broader pediatric RD literature where PPV + SB outperforms PPV alone in complex cases (88.5% vs. 56.3%; *p* = .03) [4]. Surgical technique selection should therefore be individualized by detachment type, FEVR stage, lens status, and anatomical complexity.

Outcomes in FEVR-associated RD should be contextualized against pediatric RD literature. In a large pediatric cohort by Read et al. [36], RRD had substantial postoperative improvement (mean 0.42 logMAR gain) and an 84% anatomical success rate, whereas TRD had the poorest outcomes (anatomic success 39%; only 10% achieving BCVA better than 20/200). In the FEVR subgroup, SB alone achieved the highest anatomical success (93%), far exceeding PPV (41%) or combined approaches (59%), reflecting case selection: SB was used for simpler RRD, whereas PPV and combined procedures addressed more complex detachments.

Our study has several limitations including its reliance on uncontrolled observational studies, predominantly retrospective, with inconsistent reporting of FEVR stage, surgical indications, success criteria, complications, and final visual outcomes. This heterogeneity limited subgroup analyses and increased the risk of bias. Other limitations included exclusion of non-English studies and patients under 5 years of age. Also a formal meta-regression was not performed, as most subgroups contained fewer than the recommended minimum of 10 studies per covariate. Strengths of this meta-analysis include the relatively large sample size for a rare condition, geographic diversity of studies, and access to IPD in many studies, which allowed more accurate classification by detachment and procedure type. Outcomes of RRD and TRD were analyzed separately, enabling a more nuanced understanding of prognosis and surgical strategy. Given the rarity of FEVR and the complexity of RD management, these results provide critical guidance for evidence-based decision-making.

Future research should prioritize prospective multicenter registries with standardized outcome definitions, systematic FEVR staging at baseline, and genotype-phenotype data integration — particularly given that NDP and FZD4 mutations are most strongly associated with advanced disease and RD risk [3]. The role of preoperative antiangiogenic agents in optimizing vitrectomy conditions for FEVR-TRD also warrants prospective evaluation [38, 39]. 

## Conclusion

Surgical repair of FEVR-associated RD yields high anatomical success and meaningful visual improvement, particularly in RRD. TRD outcomes remain limited despite acceptable reattachment rates, with surgical goals centered on vision preservation rather than restoration. Combined PPV + SB offers the most consistent results across complex detachments. These findings are constrained by the retrospective nature of available evidence and substantial heterogeneity.

## Supplementary Information

Below is the link to the electronic supplementary material.

Supplementary Material 1: Figure S1. Forest plot of BCVA change in rhegmatogenous retinal detachment (RRD).

Supplementary Material 2: Figure S2. Forest plot of BCVA change in tractional retinal detachment (TRD).

Supplementary Material 3: Figure S3. Forest plot of BCVA change after pars plana vitrectomy (PPV).

Supplementary Material 4: Figure S4. Leave-one-out analysis of BCVA change after PPV.

Supplementary Material 5: Figure S5. Forest plot of BCVA change after PPV for TRD.

Supplementary Material 6: Figure S6. Forest plot of BCVA change after scleral buckling (SB).

Supplementary Material 7: Figure S7. Forest plot of BCVA change after combined PPV + SB.

Supplementary Material 8: Figure S8. Forest plot of BCVA change after PPV + SB for RRD.

Supplementary Material 9: Figure S9. Leave-one-out analysis of overall RRR.

Supplementary Material 10: Figure S10. Forest plot of RRR in RRD (all surgeries).

Supplementary Material 11: Figure S11. Forest plot of RRR in TRD (all surgeries).

Supplementary Material 12: Figure S12. Leave-one-out analysis of RRR in TRD.

Supplementary Material 13: Figure S13. Forest plot of RRR after PPV.

Supplementary Material 14: Figure S14. Leave-one-out analysis of RRR after PPV.

Supplementary Material 15: Figure S15. Forest plot of RRR after PPV for TRD.

Supplementary Material 16: Figure S16. Leave-one-out analysis of RRR after PPV for TRD.

Supplementary Material 17: Figure S17. Forest plot of RRR after SB.

Supplementary Material 18: Figure S18. Forest plot of RRR after combined PPV + SB.

Supplementary Material 19: Figure S19. Forest plot of RRR in RRD treated with PPV + SB.

Supplementary Material 20: Figure S20. Funnel plot for overall BCVA.

Supplementary Material 21: Figure S21. Funnel plot for overall RRR.

Supplementary Material 22

## Data Availability

No datasets were generated or analysed during the current study.

## Declarations

### Precis

Surgical repair in familial exudative vitreoretinopathy generally achieves high anatomical success and modest average visual gains, especially for rhegmatogenous retinal detachment.
