# Supplementary figures and images for: Surgical outcomes of familial exudative vitreoretinopathy-associated retinal detachment: a systematic review and meta-analysis
Source: Int J Retina Vitreous. 2026 Apr 11;12:79. doi: 10.1186/s40942-026-00850-1 (PMC13217818; doi:10.1186/s40942-026-00850-1)

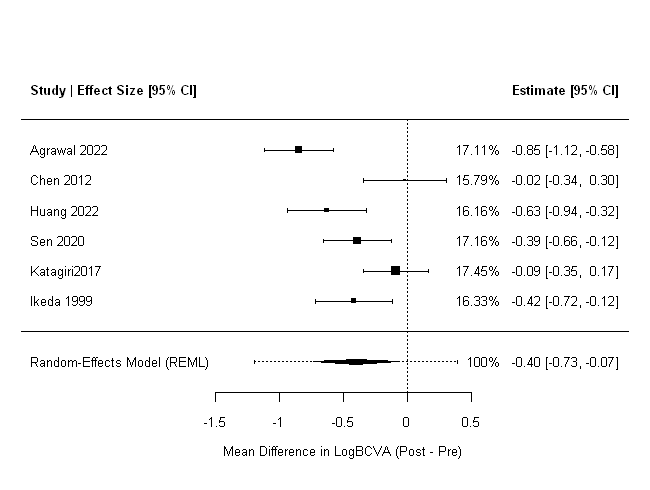

Supplement: Supplementary file 1 — Supplementary Material 1: Figure S1. Forest plot of BCVA change in rhegmatogenous retinal detachment (RRD). [file 40942_2026_850_MOESM1_ESM.tiff]

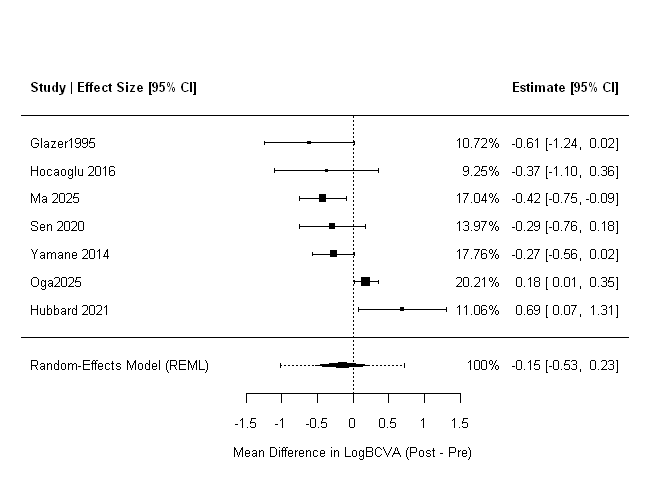

Supplement: Supplementary file 2 — Supplementary Material 2: Figure S2. Forest plot of BCVA change in tractional retinal detachment (TRD). [file 40942_2026_850_MOESM2_ESM.tiff]

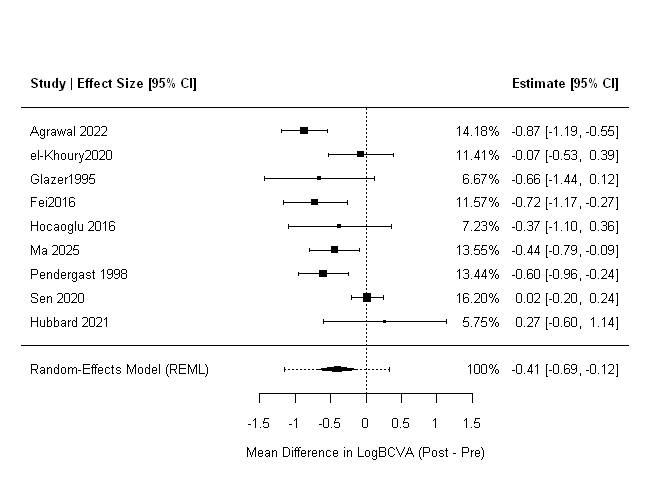

Supplement: Supplementary file 3 — Supplementary Material 3: Figure S3. Forest plot of BCVA change after pars plana vitrectomy (PPV). [file 40942_2026_850_MOESM3_ESM.tiff]

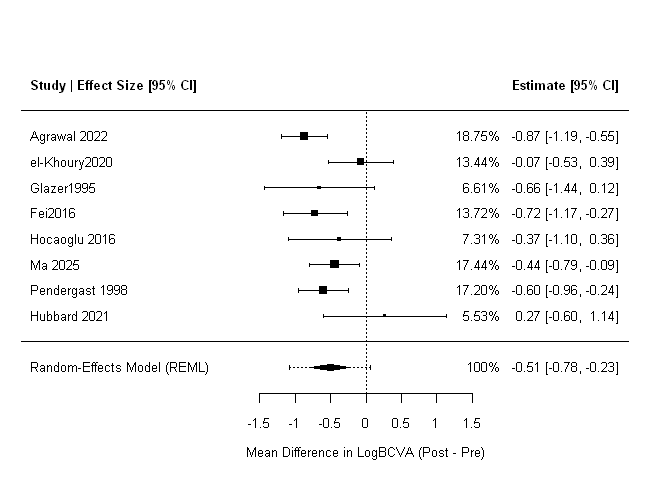

Supplement: Supplementary file 4 — Supplementary Material 4: Figure S4. Leave-one-out analysis of BCVA change after PPV. [file 40942_2026_850_MOESM4_ESM.tiff]

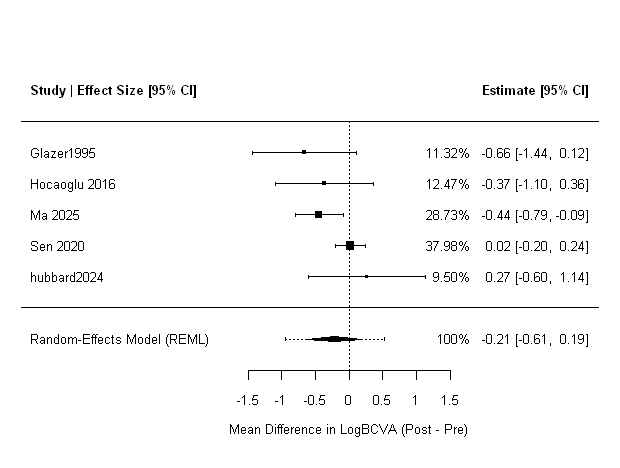

Supplement: Supplementary file 5 — Supplementary Material 5: Figure S5. Forest plot of BCVA change after PPV for TRD. [file 40942_2026_850_MOESM5_ESM.tiff]

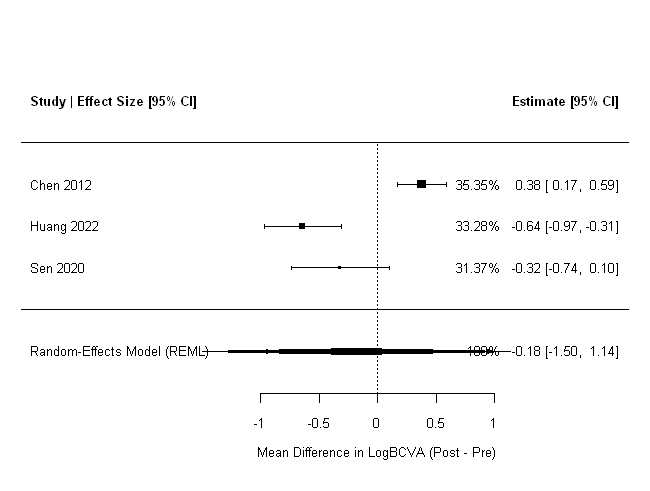

Supplement: Supplementary file 6 — Supplementary Material 6: Figure S6. Forest plot of BCVA change after scleral buckling (SB). [file 40942_2026_850_MOESM6_ESM.tiff]

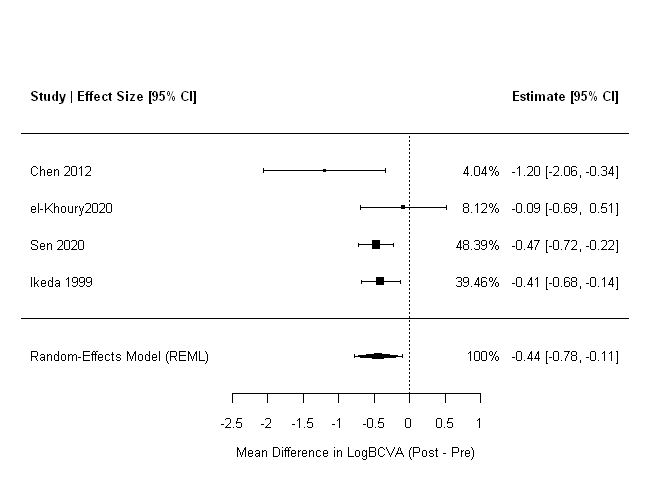

Supplement: Supplementary file 7 — Supplementary Material 7: Figure S7. Forest plot of BCVA change after combined PPV + SB. [file 40942_2026_850_MOESM7_ESM.tiff]

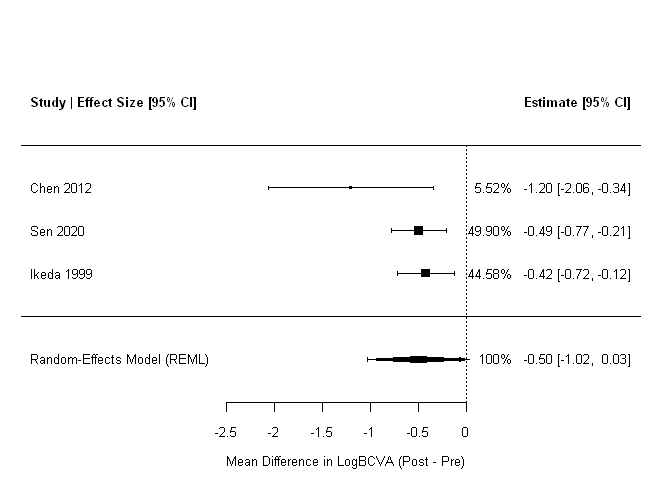

Supplement: Supplementary file 8 — Supplementary Material 8: Figure S8. Forest plot of BCVA change after PPV + SB for RRD. [file 40942_2026_850_MOESM8_ESM.tiff]

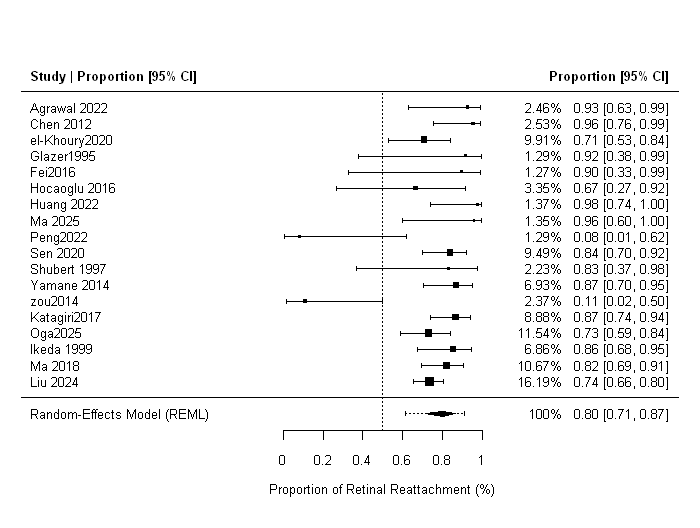

Supplement: Supplementary file 9 — Supplementary Material 9: Figure S9. Leave-one-out analysis of overall RRR. [file 40942_2026_850_MOESM9_ESM.tiff]

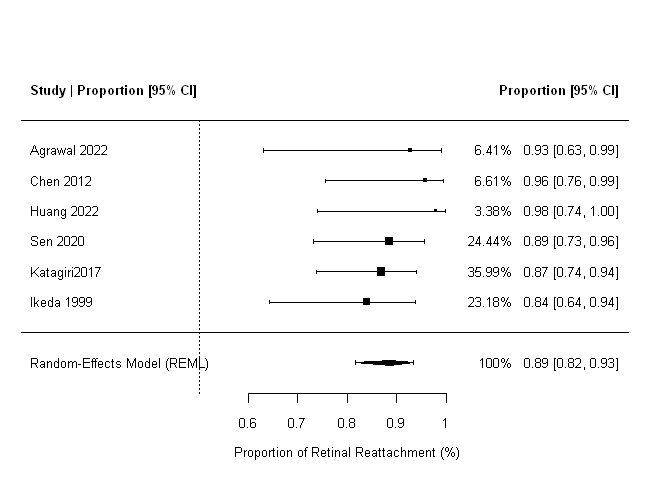

Supplement: Supplementary file 10 — Supplementary Material 10: Figure S10. Forest plot of RRR in RRD (all surgeries). [file 40942_2026_850_MOESM10_ESM.tiff]

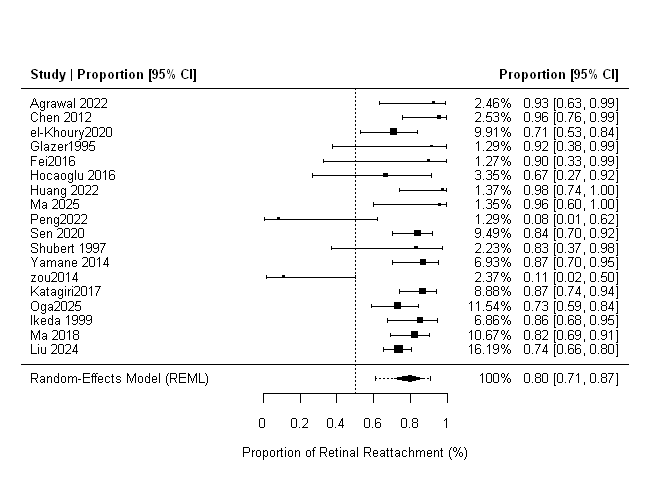

Supplement: Supplementary file 11 — Supplementary Material 11: Figure S11. Forest plot of RRR in TRD (all surgeries). [file 40942_2026_850_MOESM11_ESM.tiff]

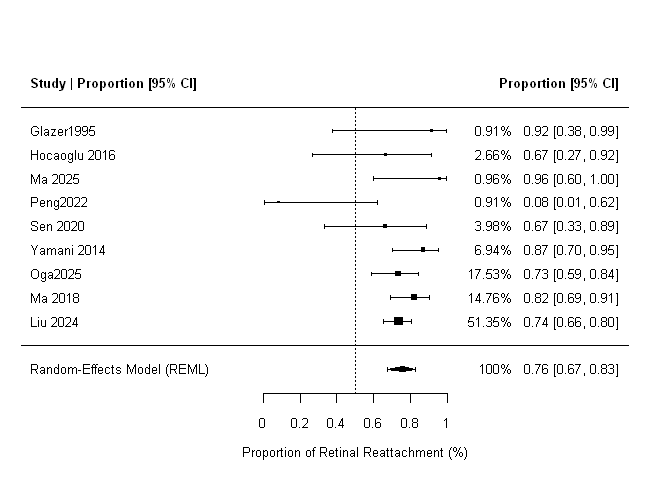

Supplement: Supplementary file 12 — Supplementary Material 12: Figure S12. Leave-one-out analysis of RRR in TRD. [file 40942_2026_850_MOESM12_ESM.tiff]

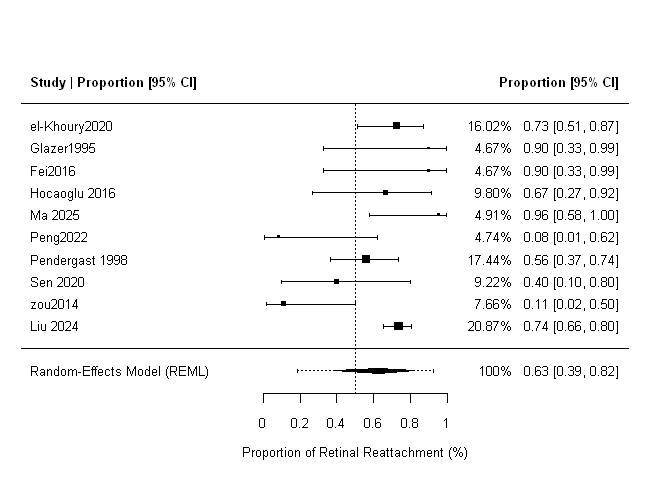

Supplement: Supplementary file 13 — Supplementary Material 13: Figure S13. Forest plot of RRR after PPV. [file 40942_2026_850_MOESM13_ESM.tiff]

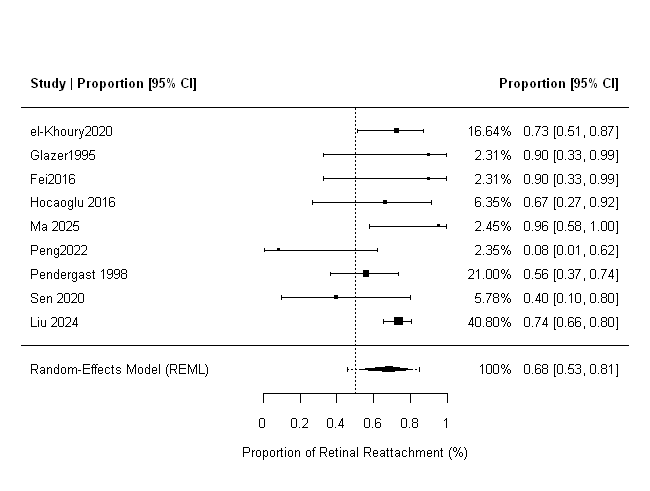

Supplement: Supplementary file 14 — Supplementary Material 14: Figure S14. Leave-one-out analysis of RRR after PPV. [file 40942_2026_850_MOESM14_ESM.tiff]

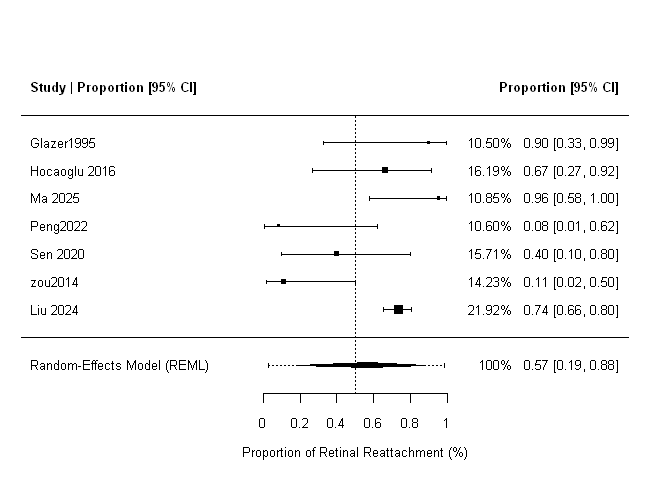

Supplement: Supplementary file 15 — Supplementary Material 15: Figure S15. Forest plot of RRR after PPV for TRD. [file 40942_2026_850_MOESM15_ESM.tiff]

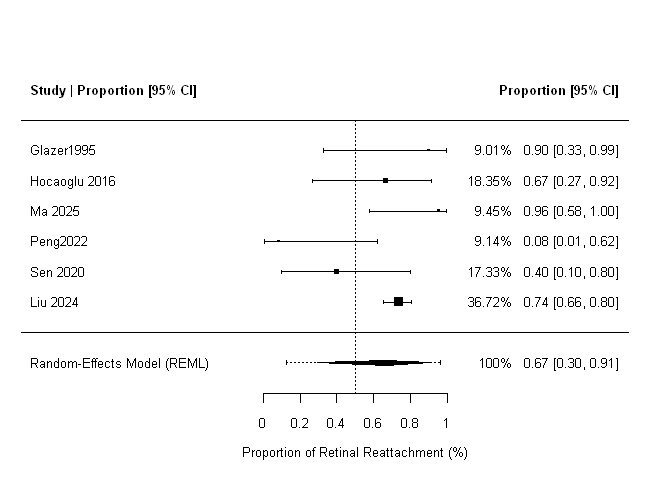

Supplement: Supplementary file 16 — Supplementary Material 16: Figure S16. Leave-one-out analysis of RRR after PPV for TRD. [file 40942_2026_850_MOESM16_ESM.tiff]

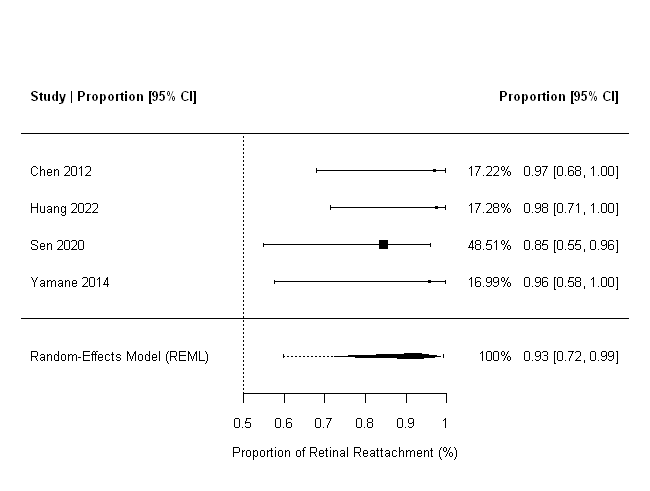

Supplement: Supplementary file 17 — Supplementary Material 17: Figure S17. Forest plot of RRR after SB. [file 40942_2026_850_MOESM17_ESM.tiff]

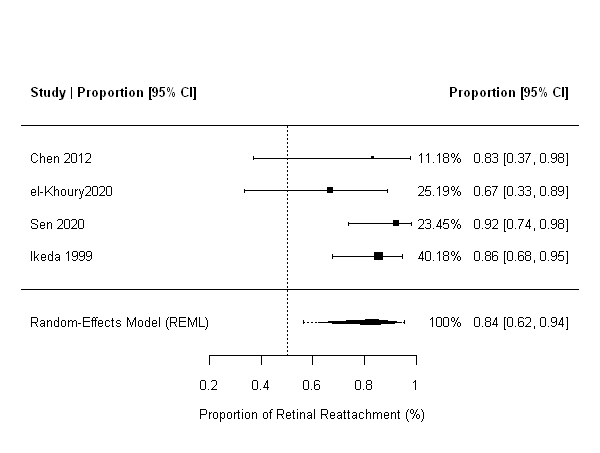

Supplement: Supplementary file 18 — Supplementary Material 18: Figure S18. Forest plot of RRR after combined PPV + SB. [file 40942_2026_850_MOESM18_ESM.tiff]

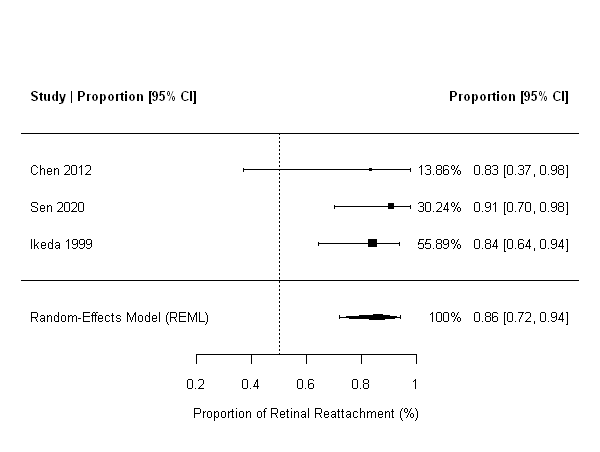

Supplement: Supplementary file 19 — Supplementary Material 19: Figure S19. Forest plot of RRR in RRD treated with PPV + SB. [file 40942_2026_850_MOESM19_ESM.tiff]

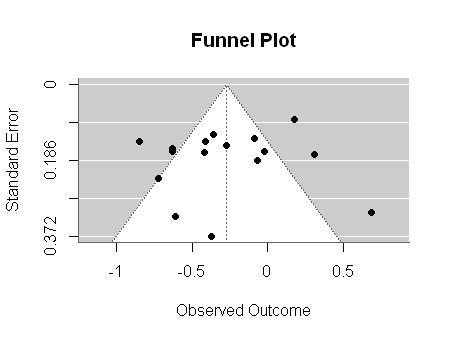

Supplement: Supplementary file 20 — Supplementary Material 20: Figure S20. Funnel plot for overall BCVA. [file 40942_2026_850_MOESM20_ESM.tiff]

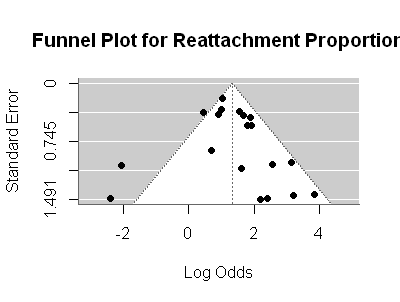

Supplement: Supplementary file 21 — Supplementary Material 21: Figure S21. Funnel plot for overall RRR. [file 40942_2026_850_MOESM21_ESM.tiff]
