# Supplementary material for: Surgical outcomes of familial exudative vitreoretinopathy-associated retinal detachment: a systematic review and meta-analysis
Source: Int J Retina Vitreous. 2026 Apr 11;12:79. doi: 10.1186/s40942-026-00850-1 (PMC13217818; doi:10.1186/s40942-026-00850-1)
Supplement: Supplementary file 22 — Supplementary Material 22 [file 40942_2026_850_MOESM22_ESM.docx]

| **Table 1. Search strategy for each database.** | | |
| --- | --- | --- |
| Database | Retrieved Results | Search Strategy (Boolean logic) |
| PubMed | 74 | ("familial exudative vitreoretinopathies"[MeSH Terms] OR "familial exudative vitreoretinopathy"[Title/Abstract] OR FEVR[Title/Abstract] OR "hereditary vitreoretinopathy"[Title/Abstract] OR "FEVR-related"[Title/Abstract]) AND ("retinal detachment"[MeSH Terms] OR "retinal detachment"[Title/Abstract] OR "rhegmatogenous retinal detachment"[Title/Abstract] OR RRD[Title/Abstract]) AND ("vitrectomy"[MeSH Terms] OR "vitrectomy"[Title/Abstract] OR "pars plana vitrectomy"[Title/Abstract] OR "PPV"[Title/Abstract] OR "scleral buckle"[Title/Abstract] OR "scleral buckling"[Title/Abstract] OR "retinal detachment surgery"[Title/Abstract] OR "retinal reattachment"[Title/Abstract] OR "retinal reattachment surgery"[Title/Abstract] OR "subretinal fluid drainage"[Title/Abstract] OR "internal tamponade"[Title/Abstract] OR "silicone oil"[Title/Abstract] OR "gas tamponade"[Title/Abstract] OR "cryotherapy"[Title/Abstract] OR "endolaser photocoagulation"[Title/Abstract]) |
| EMBASE | 100 | ("familial exudative vitreoretinopathy" OR FEVR OR "hereditary vitreoretinopathy" OR "fevr-related") AND ("retinal detachment" OR "rhegmatogenous retinal detachment" OR RRD) AND ("vitrectomy" OR "pars plana vitrectomy" OR PPV OR "scleral buckle" OR "scleral buckling" OR "retinal detachment surgery" OR "retinal reattachment" OR "retinal reattachment surgery" OR "subretinal fluid drainage" OR "internal tamponade" OR "silicone oil" OR "gas tamponade" OR cryotherapy OR "endolaser photocoagulation") |
| Web of Science | 52 | TS=("familial exudative vitreoretinopathy" OR "familial exudative vitreoretinopathies" OR "FEVR" OR "hereditary vitreoretinopathy" OR "FEVR-related") AND TS=("retinal detachment" OR "rhegmatogenous retinal detachment" OR "RRD" OR "retinal tear" OR "retinal break" OR "retina detachment") AND TS=("vitrectomy" OR "pars plana vitrectomy" OR "PPV" OR "scleral buckle" OR "scleral buckling" OR "retinal detachment surgery" OR "retinal reattachment" OR "retinal reattachment surgery" OR "subretinal fluid drainage" OR "internal tamponade" OR "silicone oil" OR "gas tamponade" OR "cryotherapy" OR "endolaser photocoagulation") |
| Scopus | 134 | TITLE-ABS-KEY("familial exudative vitreoretinopathy" OR "familial exudative vitreoretinopathies" OR "FEVR" OR "hereditary vitreoretinopathy" OR "FEVR-related") AND TITLE-ABS-KEY("retinal detachment" OR "rhegmatogenous retinal detachment" OR "RRD" OR "retinal tear" OR "retinal break" OR "retina detachment") AND TITLE-ABS-KEY("vitrectomy" OR "pars plana vitrectomy" OR "PPV" OR "scleral buckle" OR "scleral buckling" OR "retinal detachment surgery" OR "retinal reattachment" OR "retinal reattachment surgery" OR "subretinal fluid drainage" OR "internal tamponade" OR "silicone oil" OR "gas tamponade" OR "cryotherapy" OR "endolaser photocoagulation") |
| Cochrane Library | 0 | ("familial exudative vitreoretinopathy" OR FEVR OR "hereditary vitreoretinopathy" OR "fevr-related") AND ("retinal detachment" OR "rhegmatogenous retinal detachment" OR RRD) AND ("vitrectomy" OR "pars plana vitrectomy" OR PPV OR "scleral buckle" OR "scleral buckling" OR "retinal detachment surgery" OR "retinal reattachment" OR "retinal reattachment surgery" OR "subretinal fluid drainage" OR "internal tamponade" OR "silicone oil" OR "gas tamponade" OR cryotherapy OR "endolaser photocoagulation") in Title/Abstract/Keyword |
| ClinicalTrials.gov | 4 | "familial exudative vitreoretinopathy" OR FEVR OR "hereditary vitreoretinopathy" OR "fevr-related" AND "retinal detachment" AND (vitrectomy OR "pars plana vitrectomy" OR PPV OR "scleral buckle" OR "scleral buckling" OR "retinal reattachment" OR cryotherapy OR "gas tamponade" OR "silicone oil") |
| ProQuest Dissertations | 131 | ("familial exudative vitreoretinopathy" OR FEVR OR "hereditary vitreoretinopathy" OR "fevr-related") AND ("retinal detachment" OR "rhegmatogenous retinal detachment" OR RRD) AND (vitrectomy OR "pars plana vitrectomy" OR PPV OR "scleral buckle" OR "scleral buckling" OR "retinal detachment surgery" OR "retinal reattachment" OR "subretinal fluid drainage" OR "internal tamponade" OR "silicone oil" OR "gas tamponade" OR cryotherapy OR "endolaser photocoagulation") |
| Google Scholar | 200* | "familial exudative vitreoretinopathy" OR FEVR OR "hereditary vitreoretinopathy" OR "fevr-related" AND ("retinal detachment" OR "rhegmatogenous retinal detachment" OR RRD) AND (vitrectomy OR "pars plana vitrectomy" OR PPV OR "scleral buckle" OR "scleral buckling" OR "retinal reattachment" OR cryotherapy OR "silicone oil" OR "gas tamponade") |

| **Table 2.** Detailed NOS assessment for each study. | | | | | | | | | |
| --- | --- | --- | --- | --- | --- | --- | --- | --- | --- |
| **StudyID** | **Representativeness of the exposed cohort** | **Selection of the non-exposed cohort** | **Ascertainment of exposure** | **Demonstration that outcome of interest was not present at start of study** | **Control for important or additional factors** | **Assessment of outcome** | **Was follow-up long enough for outcomes to occur** | **Adequacy of follow up of cohorts** | **Total score** |
| **Agrawal2022** | 1 | 0 | 1 | 1 | 0 | 1 | 1 | 1 | 6 |
| **Chen et al., 2012** | 1 | 0 | 1 | 1 | 1 | 1 | 1 | 1 | 8 |
| **Huang et al., 2022** | 1 | 0 | 1 | 1 | 1 | 1 | 1 | 1 | 8 |
| **Katagiri2017** | 1 | 0 | 1 | 1 | 1 | 1 | 1 | 1 | 8 |
| **Fei 2016** | 1 | 0 | 1 | 1 | 1 | 1 | 1 | 1 | 8 |
| **Glazer 1995** | 1 | 0 | 1 | 1 | 0 | 1 | 1 | 1 | 7 |
| **Ma 2025** | 1 | 0 | 1 | 1 | 1 | 1 | 1 | 1 | 8 |
| **Liu 2024** | 1 | 0 | 1 | 1 | 1 | 1 | 1 | 1 | 8 |
| **Ma2018** | 1 | 0 | 1 | 1 | 1 | 1 | 1 | 1 | 8 |
| **Oga2025** | 1 | 0 | 1 | 1 | 1 | 1 | 1 | 1 | 8 |
| **Peng2022** | 1 | 0 | 1 | 1 | 0 | 1 | 1 | 1 | 6 |
| **Yamane2014** | 1 | 0 | 1 | 1 | 0 | 1 | 0 | 0 | 4 |
| **Zou2024** | 1 | 0 | 1 | 1 | 0 | 1 | 0 | 0 | 4 |
| **el-Khoury2020** | 1 | 0 | 1 | 1 | 0 | 1 | 1 | 1 | 6 |
| **Hocaoglu2016** | 1 | 0 | 1 | 1 | 0 | 1 | 0 | 0 | 4 |
| **Hubbard2021** | 1 | 1 | 1 | 1 | 0 | 1 | 1 | 1 | 7 |
| **Ikeda1999** | 1 | 0 | 1 | 1 | 0 | 1 | 0 | 0 | 4 |
| **Sen2020** | 1 | 0 | 1 | 1 | 0 | 1 | 1 | 1 | 6 |
| **Shubert1997** | 1 | 0 | 1 | 1 | 0 | 1 | 1 | 0 | 5 |
| **Pendergast1998** | 1 | 0 | 1 | 1 | 1 | 1 | 1 | 1 | 7 |
